# Factor Structure of CIWA-Ar in Alcohol Withdrawal

**DOI:** 10.1155/2014/745839

**Published:** 2014-04-06

**Authors:** Ajay Kumar Bakhla, Christoday R. J. Khess, Vijay Verma, Mahesh Hembram, Samir Kumar Praharaj, Subhas Soren

**Affiliations:** ^1^Department of Psychiatry, Rajendra Institute of Medical Sciences (RIMS), Ranchi, Jharkhand 834009, India; ^2^Department of Psychiatry, Central Institute of Psychiatry, Ranchi, Jharkhand 834006, India; ^3^Tulasi Psychiatric and Rehabilitation Centre, Mehrauli, New Delhi 110030, India; ^4^Department of Psychiatry, Ranchi Institute of Neuropsychiatry and Allied Sciences, Ranchi, Jharkhand 834006, India; ^5^Department of Psychiatry, Kasturba Medical College, Manipal, Karnataka 576104, India

## Abstract

*Objective*. To identify the underlying factor structure of alcohol withdrawal syndrome, as measured with CIWA-Ar. *Methods*. Exploratory factor analysis was conducted on the items of CIWA-Ar. On 201 alcohol-dependent male patients seeking treatment for alcohol withdrawal at 36 hours of abstinence. *Results*. A three-factor solution was obtained that accounted for 68.74% of total variance. First factor had loading from four items (34.34% variance), second factor also had four items (24.25% variance), and the third had two items (10.04% variance). *Conclusions*. Factor analysis reveals the existence of multidimensionality of alcohol withdrawal as measured with CIWA-Ar and we found three factors that can be named as delirious, autonomic and nonspecific factors.

## 1. Introduction

Alcohol withdrawal syndrome (AWS) is characterized by varied symptoms that range from mild to severe intensity depending on several factors including the quantity, frequency and duration of alcohol intake, and the number of prior withdrawal episodes, as well as individual differences in the vulnerability [1–4]. Symptoms usually present themselves within 6 to 24 hours after cessation of alcohol intake [5, 6].

Subtyping of the AWS has been attempted in the past, as Gross [7] conceptualized and proposed 3 constellations of alcohol withdrawal symptoms: factor 1 hallucinogenic that consisted of nausea, tinnitus, visual disturbance, pruritus, parasthesia, muscle pain, agitation, sleep disturbance, tactile hallucinations, and hallucinations which are auditory or visual or both; factor 2 affective and physiological that consisted of anxiety, depression, tremor, and sweats; and factor 3 delirium that consisted of clouding of the sensorium, impairment of consciousness, and impairment of contact with the observer. A cluster analytic study [8] identified three different symptoms clusters of alcohol withdrawal, namely, CNS excitation, adrenergic hyperactivity, and delirium.

Several rating instruments have been used to measure severity of alcohol withdrawal [9]. Among them, the most commonly used observer-rated scale is the 10-item clinical institute withdrawal assessment-alcohol, revised (CIWA-Ar) [10]. It has been proposed that alcohol withdrawal symptoms in CIWA-Ar appear multidimensional. A PubMed search supplemented with manual search revealed a single factor analytic study of CIWA-Ar [11]. The study by Pittman et al. [11] was to explore the relationship between AWSC and CIWA-Ar, for which they carried out study on 127 male inpatients of alcohol dependence with principle components factor extraction with varimax rotation of CIWA-Ar, a self-rated—alcohol withdrawal symptoms checklist (AWSC) and on combined items of CIWA-Ar and AWSC. They found three, five, and seven factor solution, respectively, for CIWA-Ar, AWSC, and combination of CIWA-Ar and AWSC. The analysis of CIWA-Ar was done on 7 items as 3 items had zero variance. The first factor (variance 23.9%) was “tension/anxiety” which consisted of anxiety, agitation, and tactile disturbances. The second factor (variance 22.9%) was “autonomic arousal” which consisted of paroxysmal sweats, tremor, and headache or fullness in head, whereas the third factor (variance 17.4%, eigenvalue less than 1) was “nausea and vomiting” which consisted of a single item, nausea, and vomiting.

In our setup, we use CIWA-Ar as part of the measure for the management of alcohol withdrawal symptoms. It is generally observed that alcohol withdrawal symptoms fluctuate in presentation and severity across time. The present study was carried out to explore the dimensionality of this scale in an attempt to identify a set of underlying factors that exist and can explain the interrelationships among various manifestations of acute alcohol withdrawal symptoms. The knowledge of these underlying factors may enhance our understanding of AWS and better prediction of complications thus management plans.

## 2. Materials and Methods 

### 2.1. Subjects

This was a cross-sectional hospital-based study, conducted at Centre for Addiction Psychiatry, Central Institute of Psychiatry, Ranchi, India, a tertiary care referral centre during May 2005 to June 2006. The study was approved by the institutional review board. Study sample included 201, only male fulfilling ICD-10 DCR (World Health Organization) [12] for alcohol dependence with currently withdrawal state, aged between 18 and 60 years, admitted within 24 hours of abstinence and patient himself or his guardian consenting for the study. Information on patient's demographics, treatment history, past history, and family history was obtained from interviews with patients and accompanying person. Detailed physical and neurological examinations were done to exclude any comorbid general medical condition, comorbid other psychiatric disorder, and any other comorbid substance use disorders except caffeine and tobacco.

### 2.2. Tools

*Sociodemographic Data Sheet*. The sociodemographic data sheet included age, marital status, religion, community, education, and economic status. Whereas clinical variables recorded were age of onset of drinking alcohol, duration of dependence, past history of detoxification, number of previous detoxification, past history of withdrawal seizure, past history of delirium tremens, family history of alcohol or substance dependence, degree of relationship if family history of alcohol or substance dependence is present, and family history of mental illness. 

*Clinical Institute Withdrawal Assessment of Alcohol Scale, Revised* (CIWA-Ar) [10]. It is the most widely used and studied 10-item alcohol withdrawal monitoring scale, which excludes vital sign abnormalities. It was developed from the 18-item clinical institute withdrawal assessment for alcohol and it has been studied across various geographic locations. The administration of CIWA-Ar requires approximately five minutes. It has a good reliability, validity [10], and it is considered as one of the most widely used alcohol withdrawal assessment scale for symptom-triggered therapy. Each sign and symptom item of CIWA Ar is evaluated on a 0–7 point Likert scale except for one item “orientation and clouding of sensorium”, which is scored on a 0–4 point Likert scale. The possible range of score is 0–67. A score of 8 points or less indicates mild withdrawal and patients scoring less than 10 do not usually need additional medication for withdrawal. A score of 9 to 15 points indicates moderate withdrawal. A score greater than 15 points indicates severe withdrawal.

### 2.3. Procedure

Patients admitted with alcohol dependence syndrome with acute withdrawal were evaluated with CIWA-Ar immediately after admission then every six hours, as a routine protocol of the ward. Written informed consent to participate for the study was obtained from all the patients. The sociodemographic details were obtained from patient and their relatives. Detailed physical examination, mental status examination, and planned screening laboratory investigation were done to ensure conformity of study criteria. The patients get admitted with varying lengths of abstinence, ranging from hours to days, so we initially took all the ratings of all the patients and arranged them as rating at first 6 hours of abstinence and at every six hours like at 6, 12, 18, 24, 30, 36, 42, 48, 52, 58, and 64 hours till CIWA-Ar scoring reaches below 10. The averages of each rating of CIWA-Ar scores were computed to see the severity of withdrawal symptoms across time span of abstinence. Many patients were admitted after overnight, 12 to 18 hours of abstinence, so we included the patients who were admitted with at least 24 hours of abstinence. Hence rating at the 24 hours of abstinence was considered as first rating. Meanwhile management and medications were continued as per ward protocol and no adjustment for study purpose was done. The standard detoxification protocol included thiamine supplementation, benzodiazepines either lorazepam or diazepam, and correction of fluids and electrolytes if any and other symptomatic treatment of associated conditions like dyspepsia or concurrent injury, wound, and infections.

### 2.4. Statistical Analysis

The collected data on 201 patients was statistically analyzed, using statistical package for social sciences (SPSS, Inc., Chicago, Illinois) version 10.0 for Windows. Exploratory factor analysis (maximum likelihood method) was carried out to identify factor structure on all items of CIWA-Ar for day three. Kaiser-Meyer-Olkin measure of sample adequacy and Bartlett's test of sphericity were also done to assess appropriateness of conducting factor analysis. Two criteria for retaining the number of components were considered: Kaiser's criterion [13] to retain eigenvalues greater than unity and Cattell's [14] scree plot inspection for the point of inflexion.

## 3. Results 

### 3.1. Demographics

Table 1 summarizes the sample characteristics. The mean age of the group was 37.18 (SD 9.35, range 18–69) years. Most of them were married (83.3%), educated (90%), residing in urban background (64.7%), belonging to middle socioeconomic status (60.3%), and earning a livelihood (80.4%). The mean age of starting alcohol was 21.63 (SD 4.99) years, whereas mean duration of dependence on alcohol was 6.49 (SD 5.06) years. Of these, 46.1% had past history of detoxification. 22.5% had history of withdrawal seizures, in their course of alcoholism, and only 2% reported history of delirium tremens in the past. Family history of substance dependence was present in 33.3% of the total sample, out of which alcohol dependence was present in 56.9% of subjects and cannabis dependence in 8.3%. Family history of mental illness included affective disorder in 9.8% and schizophrenia in 0.5%.

### 3.2. CIWA-Ar Score and Sample Adequacy

The item frequency and mean of all six hourly CIWA-Ar ratings were calculated; the mean scores of CIWA-Ar at 24 hours and at 36 hours are shown in Table 2. The mean CIWA-Ar score at 24 hours was 13.32 (SD 9.27) and 20.4 (SD 9.09) at 36 hours. Based on the frequency variance and total CIWA-Ar score we decided to carry out factor analysis with the 10 items of CIWA-Ar as on scoring at 36 hours of abstinence. Kaiser-Meyer-Olkin measure of sample adequacy was 0.734. Bartlett's test of sphericity was significant (*χ*2 = 1.044, df = 45, *P* < .001), indicating that a factor analysis is appropriate.

### 3.3. Factor Analysis

**Figure 1:**
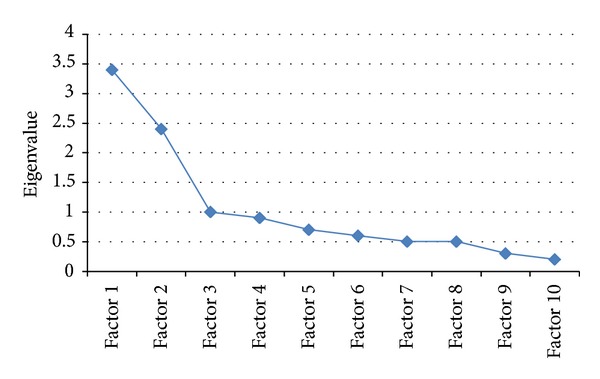
Scree plot, showing three factors above eigenvalue of one and showing clear inflexion of the graph.

Factor analysis (extraction method-maximum likelihood) with the 10 items of CIWA-Ar for day three, resulted in initial three factors with eigenvalues greater than unity. The scree plot was also showing clear inflexion, supporting three factors (Figure 1). Therefore, three factors were retained, which captured 68.74% of variance. Following varimax rotation with Kaiser's standardization, three factors were clinically interpretable. The factors and their item loadings, with absolute values greater than 0.1, are shown in Table 3. None of the items loaded on more than one factor.

The first factor named as “delirious factor,” which had highest loading from tactile disturbances (.999), followed by auditory disturbances, orientation and clouding of sensorium, and agitation. It explained 34.34% of variance and showed good internal consistency (Cronbach's alpha = .91). The second factor named as “autonomic factor” reflected four-item loading, highest from anxiety, followed by paroxysmal sweats, tremor, and headache or fullness in head. It explained 24.25% of variance and showed moderate internal consistency (Cronbach's alpha = .66). The third factor named as “nonspecific factor” reflected two-item loadings, nausea, and visual disturbances that explained 10.04% of variance and a Cronbach's alpha of .26.

## 4. Discussion 

We examined the factor structure of the CIWA-Ar in a population of adult men hospitalized to a tertiary psychiatric institute for treatment of alcohol dependence. The ideal study of AWS could have been the assessment before starting medication, but this was practically not possible for many reasons. Firstly, many of the patients come for admission after 12 to 18 hours of abstinence and severe withdrawal, so keeping them drug free was ethically not possible. Thus natural AWS presentation and its severity were masked by routine benzodiazepam administration and thiamine supplement. Secondly, many other patients were referred from primary care centers with initial management, including long acting benzodiazepines like diazepam that masks the AWS. Thirdly, many other patients came even before onset of withdrawal and in a state of intoxication; the AWS was not fully evolved in terms of range of symptoms and severity. For all these reasons, the mean CIWA-Ar score for the initial 24 hours of abstinence (first day) of admission was only 13.32 (SD 9.27). Later the sequential rating found more prominent withdrawal symptoms reaching highest mean score of 20.4 at thirty-six hours then gradually decreased. As drug free AWS was not possible, we consider that the higher mean score of CIWA-Ar represents the AWS better than low score. Also as both AWS medications and alcohol itself are CNS depressant and act in a similar way, either medication or alcohol intake should not make much difference in clinical picture. So we decided to proceed for factor analysis with highest mean CIWA-Ar scoring at the 36th hour.

The severity of withdrawal symptoms and appearance of complete sets of withdrawal symptoms at the 36th hour may have been influenced by plasma half-life of benzodiazepams being used for detoxification. In this study, choice of medication was with treating team of the hospital; however, only either intermediate acting lorazepam or long acting diazepam was used for this purpose. There was no use of short acting benzodiazepines, which causes varying and rebound withdrawal symptoms across different time frame with its dosing and changing plasma concentration. Another more important influencing reason for varying withdrawal symptoms across different time frame could have been the dose of benzodiazepines, but we did not interfered with any medications or dosing and it was continued as per ward protocol to ensure naturalistic conditions. However, the equivalent benzodiazepines mean dose at 36 hour was 30 mg of diazepam per day. But this equivalent dose may not be accurate as many patients were on oral medications and others were on parenteral benzodiazepines.

We excluded any other comorbid substance use disorders or polysubstance dependence but a few patients may also have undisclosed benzodiazepine or organic inhalant abuse or addiction. These medications with CNS depressant effect do mask and modify the withdrawal symptoms. We also excluded any comorbid general medical condition especially epilepsy for that reason, patients on antiepileptic either taking it regularly or skipping will modify the alcohol withdrawal symptoms. For that matter any psychotropic drugs causing CNS depression or any effecting stimulants will alter the withdrawal symptoms. Most of the patients needed proton pump inhibitor drugs like pantoprazole or omeprazole for the alcohol induced dyspepsia, peptic ulcer disease, or gastroesophageal reflux disease, but these medications do not impose any effect on alcohol withdrawal symptoms.

In a previous study by Pittman et al. [11], the mean CIWA-Ar score for day one was 13.2 (SD 3.7) which was similar to our study, 13.32 (SD 9.27) at 24 hours; they continued with analysis on data collected on the first study day to exclude medication effects. But we analyzed the data as of 36 hours of abstinence, showing higher mean CIWA-Ar score and all ten items had variance above 10%, thus representing fully developed withdrawal syndrome. However we could not control the medication effect used to control the withdrawal symptoms for ethical reasons, the used medications were benzodiazepine and thiamine supplementation for all the patients. Two items of CIWA-Ar, namely, auditory disturbances and tactile disturbances were very infrequent in our sample (9.5 and 10%) on day one which increased to 55.1 and 65.7% at 36 hours, other items scoring raised on day two like agitation, orientation and clouding of sensorium, visual disturbances, and paroxysmal sweats; few other items remained unchanged on day one and two like anxiety and tremor; however scoring of only this item “headache or fullness in head” improved on day two from 28.9 to 12.9% (Table 2). This indicates the importance of time duration of abstinence to study the alcohol withdrawal symptoms.

### 4.1. Factor Composition of CIWA-Ar

We found a three-factor solution based on rotated eigenvalues and scree plot analysis. The first factor explained 34.34% variance and consisted of four items, namely, tactile disturbances, auditory disturbances, orientation and clouding of sensorium, and agitation. This factor appears to represent* perceptual abnormality and delirium* and may be considered as subclinical spectrum of delirium. However, the only other factor analytic study of CIWA-Ar by Pittman et al. [11] found these items (except agitation) were infrequent in their sample and were not included in analysis. The difference may be due to the time span of withdrawal on which data was collected. The Pittman et al. [11] study analyzed CIWA-Ar for day one and also our data on day one showed very low variance for these items. This suggests that ratings on alcohol withdrawal symptoms on the very first day may miss certain set of symptoms, which appears on and around day two and are characterized by perceptual abnormality and delirium like picture. Further progression of alcohol withdrawal, we could not found beyond 36 hours, may be due to effects of continued medications for withdrawal suppression. Cronbach's alpha for this factor was 0.91 showing good internal consistency. Surprisingly, visual disturbances item did not have high loading on this factor, whereas tactile and auditory disturbances had maximum factor loading (0.999 and 0.873, resp.). The etiological basis for these two disturbances includes CNS rebound excitation which alters the perceptual quality. Furthermore, some contribution of nutritional and specific vitamin deficiencies, such as thiamine and folate, and associated peripheral neuropathy probably add to these perceptual disturbances. The other two items, orientation and clouding of sensorium and agitation with factor loadings of 0.851 and 0.777, respectively, represent delirium. The perceptual alteration and delirium being loaded in a single factor may have some predictive association and hence need to be studied for management plan.

The second “autonomic” factor explained 24.25% of variance and consisted of four items: anxiety, paroxysmal sweats, tremor, and headache and fullness in head with factor scores of .998, .660, .528, and .245 respectively. Cronbach's alpha for this factor was 0.66 showing adequate internal consistency. Within this factor the highest loadings was with anxiety. It probably represents mixed mechanism of CNS rebound hyperactivity along with adrenal hyperactivity. This factor may be a result of the practice of not using adrenergic medication on routine basis at our institute. This factor was in accordance to study of Pittman et al. [11] and all items (paroxysmal sweats, tremor, and headache or fullness in head) were loaded similarly. We had additional anxiety to this factor; hence, we share the name of this factor with Pittman et al. [11] as “*autonomic*”. The third “nonspecific” factor explained 10.04% of variance and consisted of two items: nausea and vomiting and visual disturbances, with low factor scores of .51 and .27, respectively. The Cronbach alpha for this factor was 0.26 showing poor internal consistency. These two items represent some mixed rather than any specific mechanism.

There are three proposed physiologic bases for the symptom manifestation of alcohol withdrawal symptoms: CNS excitation, adrenergic hyperactivity, and delirium, which may be attributed to different neurotransmitters and they respond selectively to different pharmacotherapy [8]. The CNS excitation may be secondary to deficiency in GABA activity [15], whereas increase in CNS epinephrine level causes adrenergic hyperactivity. The NMDA receptor hypersensitivity and overactivity of certain subtypes of NMDA receptors are associated with delirium [16]. Though we did not find a factor structure of AWS in accordance with very strict pathophysiological manifestation of either autonomic, or CNS excitation or psychological/affective, or perceptual/hallucinogenic in our sample, the obtained factors suggest existence of subgroups of patients with different set of symptoms. This was expected as alcohol withdrawal manifests by simultaneous involvement of all mechanisms rather than any mutually exclusive mechanism. This simultaneous involvement of several other neurotransmitters besides GABA and NMDA, like noradrenaline, acetylcholine, and dopamine as well as hormones and electrolytes [17, 18] will affect the symptom presentation. Additionally our data analysis had CIWA-Ar ratings of day two (36 hours) of admission with suppression of withdrawal symptoms from detoxification medications, that is, benzodiazepines. This would have differential effect on withdrawal symptoms manifestation in terms of GABA suppression only and selective unopposed action on other neurotransmitters or lacking adrenergic activity. Though avoiding pharmacological suppression or modification was not possible on ethical grounds to understand completely natural unmodified withdrawal manifestation. There may also be possible influence of different types of alcoholic beverage and percent content of alcohol.

Strength of our study includes large sample size and not interfering with any medications or management strategies thus providing setting of naturalistic conditions. The use CIWA-Ar is the most widely accepted alcohol withdrawal assessment scale and selection of abstinent hours was important to allow time for full appearance of symptoms, even though under cover of detoxification medication. There was for better coverage and inclusion of withdrawal symptoms at 36 hours as indicated by total CIWA-Ar score and item frequency.

These results have wider implications for the recognition and management of AWS, particularly, for better understanding and identification of the symptom profiles for and differential management plans across subtypes of AWS. The use of adrenergic antagonists may have a valuable role in addition to benzodiazepines, in a set of patients with autonomic features. One of the limitations in our study is that it includes male only patients; however, gender can be an important issue in AWS presentation and its severity. Even studies found that sex hormone affects the AWS by modulating the function of the GABA-A receptor [19]. Also low levels of testosterone are associated with symptoms like indecision, excessive worrying, fatigability, and lassitude [20]. Another limitation of this study is patients sample with severe AWS requiring inpatient management, thus limiting generalizability of our findings across gender and to mild to moderate severity cases. Another limitation was that diagnosis was made clinically with ICD-10 DCR criteria for “alcohol dependence with currently withdrawal state”, but not by a validated clinical interview.

It is known that autonomic arousal is an important mechanism in AWS; thus, other physiological measures and biological markers for objective assessment may be included in future studies. Further studies may also be carried out including cases of mild to moderate severity and in both sex to uncover the differences. Also, factor analysis depends heavily on the population studied; therefore, studies on different population may be required to generalize our findings. For the clinical practice, it is advisable not to overdepend on rating scales and it must not replace a thorough clinical evaluation of the patient's medical status in prediction of those at risk of severe alcohol withdrawal.

## 5. Conclusion

The acute alcohol withdrawal symptoms was most severe at 36 hours of abstinence in our sample. This study finds multidimensionality of alcohol withdrawal symptoms as measured with CIWA-Ar; we found three factors explaining 68.74 percentage of variance and named as delirious, autonomic and nonspecific. These factors of the CIWA-Ar represent high internal consistency among the items.

## Figures and Tables

**Table 1:** Sample characteristics (*N* = 201).

Variables	Mean	SD
Age	37.18	9.35
Age of onset of drinking (in years)	21.63	4.99
Duration of dependence (in years)	6.49	5.06
Average amount (in mL)	1266.59	870.85
Maximum amount (in mL)	1802.36	1191.72
Last intake (hours before admission)	13.64	9.27

Variables	*N *	%

Gender		
Male	201	100
Marital status		
Married	170	84.6
Single	31	15.4
Education		
Illiterate	18	9.0
Up to class 10	71	35.3
Above class 10	112	55.7
Habitat		
Rural	25	12.4
Urban	132	65.6
Semiurban	44	22.0
Occupation		
Unemployed	34	17.0
Employed	167	83.0
Socioeconomic status (monthly income in Rs)		
Lower (upto 5000)	68	34.0
Middle (5000–20000)	123	61.0
Higher (above 20000)	10	5.0
Past history of detoxification	94	46.8
Past history of withdrawal seizure	46	22.9
Past history of delirium tremens	4	2.0
Family psychiatric illness	24	12.0
Family substance dependence	133	66.2

**Table 2:** CIWA-Ar items frequency, mean, and SD at 24 hours and 36 hours (*N* = 201)*.

CIWA-Ar items	24 hours			36 hours		
	Present%	Mean	SD	Present%	Mean	SD
(1) Anxiety	91	3.67	2.06	91	2.96	1.92
(2) Nausea	25.9	.51	1.09	58.7	.92	.95
(3) Paroxysmal sweats	69.2	1.31	1.54	75	1.04	.86
(4) Headache and fullness in head	28.9	.44	.86	12.9	.17	.49
(5) Tremor	94.5	3.82	1.99	94.5	4.62	1.78
(6) Visual disturbances	30.3	1.01	1.75	53.2	.72	.90
(7) Auditory disturbances	9.5	.29	1.38	55.1	3.35	2.54
(8) Tactile disturbances	10	.30	1.33	65.7	2.25	2.20
(9) Orientation and clouding of sensorium	11	.29	.95	49.3	1.05	1.30
(10) Agitation	65.7	1.63	1.99	80.6	3.30	2.06

Total CIWA-Ar score	13.32 ± 9.27			20.4 ± 9.09		

*based on this table, we decided to conduct factor analysis on the data of 36 hours of abstinence.

**Table 3:** Factor analysis (maximum likehood) with varimax rotation showing factor structure of CIWA-Ar (*N* = 201) at 36 hours.

CIWA-Ar items	Factor loading			% positive	Item scores	
	Delirious	Autonomic	Nonspecific		Mean	SD
Tactile disturbances	**.999**			65.7	2.26	2.20
Auditory disturbances	**.873**	.150	.163	75.1	3.35	2.54
Orientation and clouding of sensorium	**.851**		−.137	49.3	1.06	1.30
Agitation	**.777**	.120		80	3.30	2.06
Anxiety		**.998**		91	2.96	1.92
Paroxysmal sweats		**.660**	.277	75	1.05	.86
Tremor		**.528**	.211	94.5	4.63	1.78
Headache and fullness in head	.172	**.245**		12.9	.18	.49
Nausea and vomiting	−.156	.375	**.516**	58.7	.93	.95
Visual disturbances	.233		**.275**	53.2	.72	.90
Eigenvalue	3.4	2.4	1			
Variance (%)	34.34	24.25	10.04		Total = **68.74**	
Factor mean (SD)	9.97 (7.4)	8.81 (3.96)	1.64 (1.41)			
Cronbach's alpha	.91	.66	.26			
